# The influence of probiotics on genital high-risk human papilloma virus clearance and quality of cervical smear: a randomized placebo-controlled trial

**DOI:** 10.1186/s12905-019-0798-y

**Published:** 2019-07-24

**Authors:** Yu-Che Ou, Hung-Chun Fu, Chih-Wen Tseng, Chen-Hsuan Wu, Ching-Chou Tsai, Hao Lin

**Affiliations:** 1 Department of Obstetrics and Gynecology, Chia-Yi Chang Gung Memorial Hospital, Chia-Yi, Taiwan; 2 Department of Obstetrics and Gynecology, Kaohsiung Chang Gung Memorial Hospital and Chang Gung University College of Medicine, 123, Ta Pei Road, Niao Sung District, Kaohsiung City, 83301 Taiwan, Republic of China

**Keywords:** *Lactobacillus rhamnosus*, *Lactobacillus reuteri*, Human papilloma virus, Cervical smear

## Abstract

**Background:**

Probiotics has been shown to be effective in reducing vaginal colonization of pathogenic organisms. The aim of this study was to investigate the influence of probiotic strains *Lactobacillus rhamnosus* GR-1 and *Lactobacillus reuteri* RC-14 on genital high-risk human papilloma virus (HR-HPV) clearance and quality of cervical smear.

**Methods:**

This was a randomized, double-blinded, placebo-controlled trial. Women with genital HR-HPV infection were randomized into study and control groups. A probiotic or placebo preparation was administered orally (one capsule daily) until negative HR-HPV testing. A cervical smear and HR-HPV tests were performed at the beginning of the study and every 3 months thereafter until a negative result was obtained.

**Results:**

A total of 121 women with genital HR-HPV infection were enrolled (62 in the study group and 59 in the control group). There was no significant difference in HR-HPV clearance rate between the two groups (58.1% vs. 54.2%). The only factor predicting HR-HPV clearance was a lower initial viral load (HR 3.214; 95% CI: 1.398, 7.392; *p* = 0.006). Twenty-two women had a mildly abnormal initial cervical smear and nine had an unsatisfactory smear. At 6 months follow-up, both mildly abnormal cervical smear and unsatisfactory smear rates had decreased significantly in the study group compared to the control group (*p* = 0.017 and 0.027).

**Conclusions:**

The application of probiotic strains *Lactobacillus rhamnosus* GR-1 and *Lactobacillus reuteri* RC-14 did not influence genital HR-HPV clearance, but may have decreased the rates of mildly abnormal and unsatisfactory cervical smears.

**Trial registration:**

Clinicaltrials.gov NCT01599416, May, 2012. Retrospectively registered.

## Background

A healthy vaginal microbiome, mostly containing lactobacilli microorganisms, can aid in the prevention of bacterial vaginosis (BV), fungal infections and other possible problems by maintaining an acidic pH (< 4.5) that is unfavorable for the growth of common pathogens. The reduction or absence of vaginal lactobacilli has been shown to be a major cause of BV, and to increase the risks of vaginitis and recurrent urinary tract infections by 2–4 times [1, 2]. In addition, harmful bacteria transmitted via sex or an imbalance in bacteria may cause a change in pH in the vagina, thereby presenting a suitable environment for virus infection and expansion [3, 4]. Furthermore, a positive association between BV and cervical high-risk human papilloma virus (HR-HPV) infection was suggested in a meta-analysis by Gillet et al. [5].

Epidemiologic studies have indicated that HR-HPV is the main etiological agent in the development of cervical cancer. However, only 10% of HR-HPV infections persist and potentially progress to cervical cancer [6]. It is unknown why HR-HPV infection is cancerous in some women but eradicated in others. Individual differences in immunological defense may be one explanation, and local cervical factors may also determine the outcome of HR-HPV infection [6, 7].

The quality and sensitivity of a cervical cytological diagnosis can be affected by the presence of vaginal infections due to the accumulation of numerous microorganisms, white blood cells, and degradation products. In order to improve the reliability of cervical cytology, treatment of vaginal infections and maintaining a healthy vaginal environment are necessary. U-relax® (U-relax, Tri-factor Biotech Inc., Taiwan), an oral probiotic used to restore vaginal flora, contains two patented and clinically proven probiotic strains: *Lactobacillus rhamnosus* GR-1 and *Lactobacillus reuteri* RC-14. Reid et al. had reported evidence that these two probiotic strains can be delivered to the vagina following oral intake via morphology identification and molecular typing [8]. Clinically, oral U-relax® has been shown to be safe and effective in reducing vaginal colonization of pathogenic bacteria and yeast [9–11]. However, the association between these probiotics and HR-HPV infection has not been fully investigated. Therefore in present study, we attempted to evaluate the influence of oral U-relax® on genital HR-HPV clearance and quality of cervical smear diagnosis.

## Methods

The study was a randomized, double-blinded, placebo-controlled clinical trial. Participants were recruited at Kaohsiung Chang Gung Memorial Hospital from January 2010 through June 2013. After Institutional Review Board approval, informed consent was obtained from all patients. The inclusion criteria were 1) females aged 30 to 65 years with HR-HPV infection, 2) cervical smear test with negative for intraepithelial lesion or malignancy results, 3) not pregnant. The exclusion criteria included 1) cervical cancer patients, 2) cervical intraepithelial neoplasia before conization, and within 6 months after conization, 3) gastrointestinal dysfunction or prior history of gastrointestinal surgery. All women received cervical smears and HR-HPV testing to confirm the HR-HPV infection. A colposcopic biopsy was performed in patients with cervical cytology of ASCUS and above.

In order to achieve 75% power at a 0.05 significance level, an overall sample size of at least 180 subjects (90 in the control and 90 in the treatment group) should be enrolled. However, enrollment was slow therefore a decision of closing accrual was made 2 years after study commenced. Randomization was performed through application program provided at the website (http://www.randomization.com/) using a computerized, balanced (1:1) method. Random numbers were generated by a computer, and the randomization code was inserted into numbered, sealed, opaque envelopes. A single envelope was opened by the patient when they were included. Study coordinators, patients, gynecologists and members of the panel were masked to the intervention after assignment. The study group was treated orally with one capsule a day of U-relax® (U-relax, Tri-factor Biotech Inc., Taiwan). Each capsule contains 180 mg of a standardized, light beige fine powder (glucose anhydrate, potato starch, microcrystalline cellulose and magnesium stearate) consisting of freeze-dried cultures (50% *Lactobacillus rhamnosus* GR-1 and 50% *Lactobacillus reuteri* RC-14). Quality control on the product showed that each capsule of U-relax has a minimum potency of 5.4 billion (5.4E+ 9) CFU (Colony Forming Units) and can be stored in room temperature 5-30C without dramatic change in CFU. The control group received the same capsule contains 180 mg of a standardized, light beige fine powder but without any probiotic bacteria. The treatment was discontinued until negative HR-HPV testing.

Cervical swabs from the uterine cervix and endocervix were obtained every 3 months for cytology examinations and also for HR-HPV testing until a negative result was obtained. For cervical cytology, the swab was transferred to a glass microscope slide glass, fixed in 95% ethanol and tinted using Papanicolaou (hematoxylin, methyl orange, and polychrome). The preparations were then analyzed for abnormal cells by cytopathologists, and the results were classified according to the Bethesda System. Inflammation was defined as an increased number of polymorphonuclear leukocytes or neutrophils and parabasal cells with generalized eosinophilia of the cells. Unsatisfactory result was defined as more than 75% of the epithelial cells were obscured or could not be clearly visualized. For HR-HPV testing, we used Hybrid Capture 2 (HC2) test kits (Digene, Silver Spring, MD, USA) according to the manufacturer’s protocol. Samples with a relative light unit (RLU) ratio higher than 1.0 were recorded as positive, and viral load was defined as RLU/positive controls.

Age, parity, menopausal status, educational level, employment status, prior hysterectomy, intrauterine device (IUD) use, viral load, HPV clearance, and cervical smear results were compared between the two groups. Data obtained from this study were analyzed using the chi-square test, Fisher’s exact test and Student’s *t*-test. Hazard ratios (HRs) were estimated from multivariate logistic regression models to identify the independent factors predicting HPV clearance. The Kaplan-Meier method was used to estimate the time to clearance of HPV infection. All data were analyzed using SPSS statistical software version 22 (IBM Institute, Cary, NC, USA). Results with a *p* value < 0.05 were considered to be statistically significant.

## Results

**Fig. 1:**
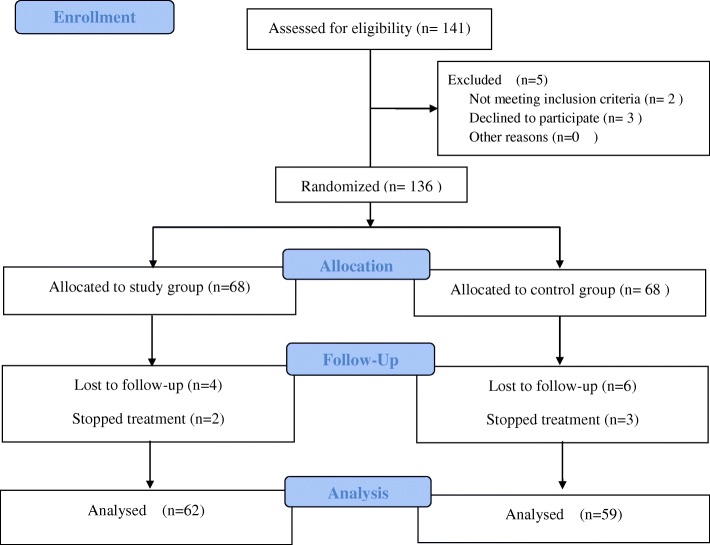
Consort flow diagram

Finally a total of 141 women were assessed for eligibility. Five were excluded due to not meeting inclusion criteria or declined to participate, and the remaining 136 women with HR-HPV infection were enrolled and randomly allocated to the study (68 women) and control (68 women) groups. A two-sided log rank test achieved 55.8% power at this sample size. During followed up period, 6 and 9 women in the study and control groups, respectively, were further excluded for analysis due to lost follow-up or stopped treatment. The CONSORT flow diagram is shown in Fig. 1. The patients’ demographic data are summarized in Table 1. There were no significant differences in age, parity, menopausal status, educational level, employment status, prior total hysterectomy history, intrauterine device (IUD) use, pretreatment viral load, and HR-HPV clearance between the study and control groups.

**Table 1:** Demographic characteristics of the study and control groups

	Study Group *N* = 62	Control Group *N* = 59	*p*-value
Age, years (mean ± SD)	45.81 ± 10.83	43.83 ± 11.06	0.897
Post-menopause	18 (29.0%)	19 (32.2%)	0.839
Parity	1.91 ± 1.05	2.12 ± 1.23	0.327
IUD	8 (12.9%)	4 (6.7%)	0.268
Educational level (college or above)	22 (35.5%)	17 (28.8%)	0.652
Employed	42 (67.7%)	36 (61.0%)	0.559
History of TH	4 (6.5%)	7 (11.9%)	0.355
HR-HPV load (RLU/PC) (mean ± SD)	427.08 ± 771.50	416.06 ± 719.51	0.686
HR-HPV clearance	36 (58.1%)	32 (54.2%)	0.716
ASCUS/LSIL	13 (21.0%)	9 (15.3%)	0.522

*ASCUS/LSIL* atypical squamous cell of undetermined significance/low-grade squamous intraepithelial lesion, *HR-HPV* high-risk human papilloma virus, *IUD* intra-uterine device, *RLU/PC* relative light unit/positive control, *SD* standard deviation, *TH* total hysterectomy

**Fig. 2:**
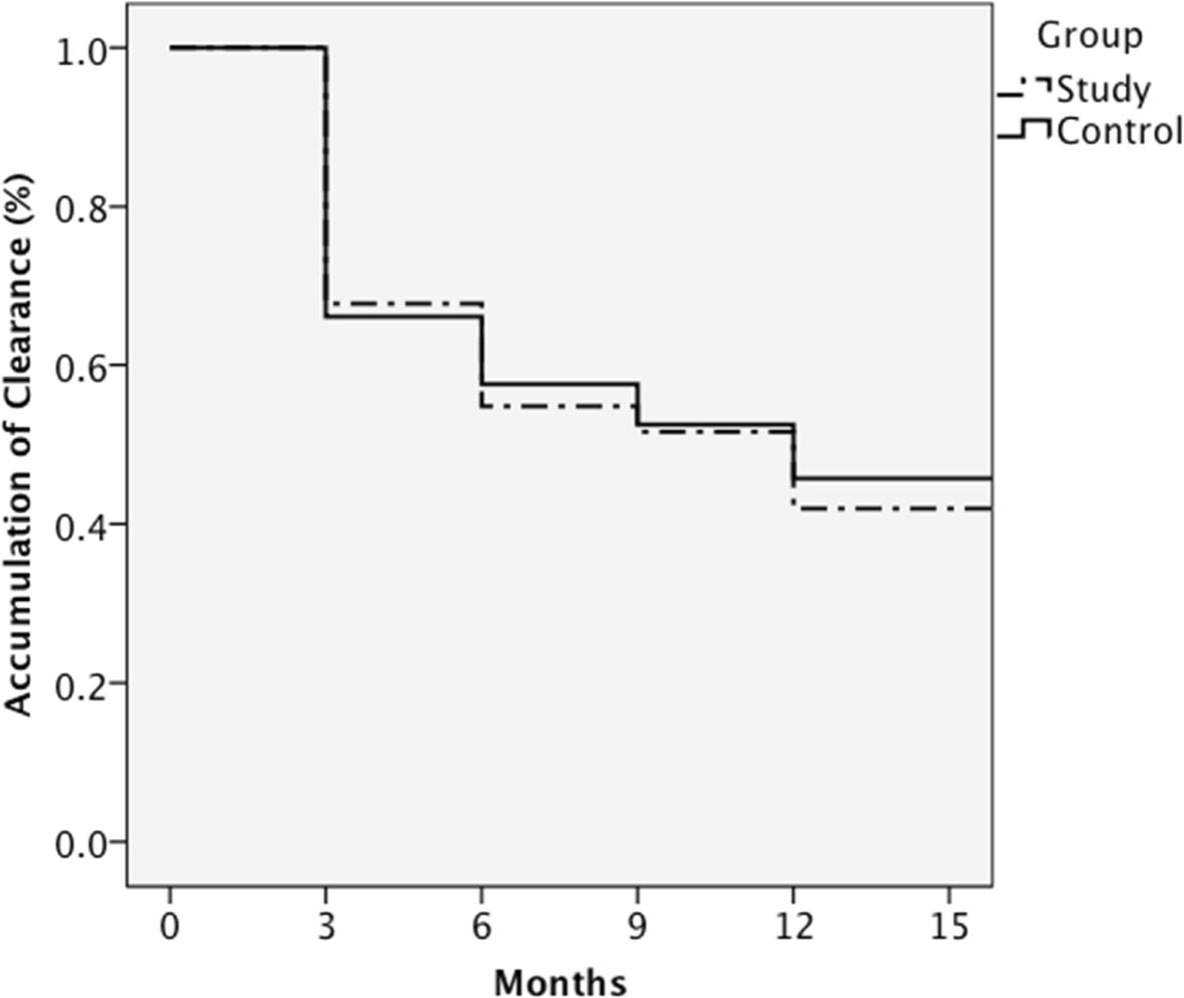
Time to HR-HPV clearance; there was no significant difference between the two groups (log rank test, *p* = 0.741)

With regards to viral clearance, HR-HPV was cleared in 33.1, 43.8, 47.9, and 56.2% of the entire cohort at 3, 6, 9, and 12 months follow-up, respectively. The clearance rate was similar between the study (58.1%) and control (54.2%) groups. Figure 2 shows the Kaplan-Meier curves of the two groups for the estimated time to clearance of HR-HPV infection. There was no difference in the time to clearance between the two groups (log rank test, *p* = 0.741). Age, menopausal status, educational level, employment status, prior hysterectomy, intrauterine device (IUD) use, and the use of probiotics were not associated with viral clearance. However, the women with HR-HPV clearance had significantly lower mean parity (*p* = 0.03) and lower initial viral load (*p* = 0.019). In multivariate logistic regression analysis, a lower initial HR-HPV viral load was the only independent factor predicting viral clearance after adjusting for confounding factors (HR 3.214, 95% confidence interval: 1.398, 7.392; *p* = 0.006) (Table 2).

**Table 2:** Univariate and multivariate analyses of factors predicting HR-HPV clearance

	HR-HPV clearance			Multivariate analysis	
	Yes (*n* = 68)	No (*n* = 53)	*p*-value	HR (95% CI)	*p*-value
Age, years (mean ± SD)	44.72 ± 10.50	45.00 ± 11.58	0.890	0.494 (0.092, 2.654)	0.623
Post-menopause	21 (30.9%)	16 (30.2%)	0.986	1.007 (0.451, 2.251)	0.848
Parity	1.81 ± 1.01	2.28 ± 1.25	0.030	2.100 (0.949, 4.648)	0.105
IUD	8 (11.8%)	4 (7.6%)	0.752	1.469 (0.405, 5.326)	0.734
Educational level (College or above)	25 (36.7%)	14 (26.4%)	0.310	1.637 (0.725, 3.693)	0.440
Employed	44 (64.7%)	34 (64.1%)	0.846	1.107 (0.513, 2.386)	0.449
History of TH	4 (5.9%)	7 (13.2%)	0.104	0.260 (0.065, 1.032)	0.147
Probiotics used	36 (52.9%)	26 (49.1%)	0.716	1.168 (0.569, 397)	0.672
Viral load (RLU/PC) (mean ± SD)	313.82 ± 616.28	561.88 ± 868.58	0.019	3.214 (1.398, 7.392)	0.006

*CI* confidence interval, *HR* hazard ratio, *HR-HPV* high-risk human papillomavirus, *IUD* intra-uterine device, *RLU/PC* relative light unit/positive control, *SD* standard deviation, *TH* total hysterectomy

Cervical cytological findings before and after therapy are presented in Table 3. Overall, 22 women initially had a mildly abnormal cytological diagnosis (11 ASCUS and 11 LSILs), and nine women had unsatisfactory results. The unsatisfactory rate was rather high in our study, mostly due to excessive inflammatory cells rather than inadequate sampling. There were no significant differences between the study and control groups before treatment in terms of inflammation, ASCUS, LSILs, and unsatisfactory results. There were no significant changes in the cytological findings between the two groups at 3 months follow-up. However, at 6 months follow-up, only 10 and 6 women had a mildly abnormal cytology and unsatisfactory results, respectively. There were a significant decrease in ASCUS/LSILs in the study group (13 to 4) compared to the control group (9 to 6) (*p* = 0.017). The unsatisfactory cases also dramatically dropped from 5 to 2 in the study group while there was no change in the control group (*p* = 0.027). The cytological findings at 9 and 12 months remained similar between the two groups. No progression of cytological results was noted throughout the study period.

**Table 3:** Distribution of cervical smear results before and after probiotic treatment between the two groups

	Study Group (*N* = 62)		Control Group (*N* = 59)		
	Pre-Treatment	Post-Treatment (6 months)	Pre-Treatment	Post-Treatment (6 months)	*p*-value
Normal	13 (20.9%)	26 (41.9%)	18 (30.5%)	23 (39.9%)	0.672
Inflammation	31 (50%)	30 (41.9%)	28 (47.4%)	26 (44.1%)	0.365
ASCUS and LSIL	13 (21.0%)	4 (6.5%)	9 (15.3%)	6 (10.2%)	0.017
Unsatisfactory	5 (8.1%)	2 (3.2%)	4 (6.8%)	4 (6.8%)	0.027

*ASCUS* atypical squamous cells of undetermined significance, *LSIL* low-grade squamous intraepithelial lesion

## Discussion

The rationale of using probiotic strains in HPV clearance is via three proposed mechanisms. First, a synergistic environment is created. The anti-microorganism effects of probiotic strains are through space competition, nutrition competition, and the production of inhibitory compounds (biosurfactants, hydrogen peroxide, lactic acid, bacteriocins, and coaggregation molecules) [12]. Thus an increased number of probiotic strains in the vagina may be able to prevent and reduce HPV infections or expansion. Second, enhanced innate and adaptive immunity, which is the major defense mechanism against viral infections. Many studies have reported that immunization with the probiotics GR-1 and RC-14 can increase CD4 count in patients with acquired immune deficiency syndrome (AIDS) [13], and regulate TNF (tumor necrosis factor)-alpha, IL (interleukin)-6, IL-8, IL-10 and IL-12 (p70) in the neurogenic bladders of patients with spinal cord injuries with urinary tract infections [14]. Third, through a direct antiviral effect via the secretion of specific metabolites [15]. Cadieux et al. found that probiotic strains had the ability to inactivate viruses [16]. Cha et al. also showed an antiviral activity on HPV type 16 through suppression of E6 and E7 oncogene expression in vitro [17]. The potent antiviral activity may in part explain the reduced risk of women acquiring sexually transmitted diseases, including HIV.

Two studies investigating the association between probiotics and HPV had been reported. In a recent trial, Palma et al. reported that HPV clearance was higher in the treatment of metronidazole plus 6 months vaginal Lactobacillus implementation than that with 3 months use, no control arm was included in that trial [18]. However, in a previous randomized pilot study, Verhoeven et al. failed to find any influence of probiotics on HPV clearance in a group of women with HPV-related LSILs of using oral Lactobacillus casei Shirota (1X10^10^CFU/day) for 6 months [19]. Our report is the third study to evaluate the efficacy of probiotics on HR-HPV clearance. The population investigated in our study was somewhat different to that of Verhoeven et al., in that only 11% of our patients with HR-HPV infection had LSILs. We also failed to demonstrate an association between the use of probiotic strains and genital HR-HPV clearance. Although the findings of these studies have tended to be contradictory, there are still several issues that need to be clarified such as the dosage and duration of probiotic treatment, the route of administration, and the effect of probiotics on different types of HPV. Therefore, whether the role of probiotics in vaginal HPV infection is preventive, therapeutic, or both still requires further investigation.

Cervical smear screening is an effective method to detect cytological abnormalities. However, the quality of a smear is sometimes compromised by inflammatory cells and exudate, inadequate cellularity or failure to sample the transformation zone leading to an unsatisfactory result. A high unsatisfactory rate can increase the number of patient revisits, thereby increasing the overall cost of the screening program. Therefore how to lower the unsatisfactory rate has become an important issue before adopting cervical smear testing protocols into a large-scale screening program. The use of liquid-based cytology has been shown to be effective in reducing the unsatisfactory rate compared with conventional smears as it provides a cleaner smear though removing obscuring elements such as blood and inflammation [20]. The reduction has been shown to be greatest in younger women and to decrease with increasing age [21]. The reason may be due to lower cellularity with tissue atrophy in a non-estrogenic state following reduced ovarian function, which cannot be overcome by liquid base cytology [22]. It has been well established that probiotic strains can enable restoration and maintenance of normal vaginal flora and are thus helpful in the treatment and prevention of BV and vulvovaginal candidiasis [8, 9]. This effect has also been shown in post-menopausal women [23, 24]. A previous study showed that number of lactobacillus was increased following probiotic administration in postmenopausal women indicating low estrogen levels wound not influence probiotic efficacy [25]. However whether the restoration of normal flora can increase cervical smear cellularity remains unknown. Therefore, the influence of probiotics on cervical smear quality is an interesting topic. Nevertheless, we only identified one related study in a literature search. Perisic et al. reported that the use of *Lactobacillus rhamnosus* GR-1 and *Lactobacillus reuteri* RC-14 could decrease the presence of unsatisfactory and/or borderline satisfactory cytological findings and thus provide a more reliable cytological diagnosis [26]. In our hospital, the annual Pap smear unsatisfactory rate is around 2.5%, however in the present study the rate was as high as 7.44%. There are two possible reasons for this finding. First, all cervical smears were obtained from conventional methods, and second, all of the participants had HR-HPV infections and BV was independently associated with HPV infection [5]. The high unsatisfactory rates in both arms just make our research feasible. We found a significant decrease in the unsatisfactory rate in the study arm, indicating the possible role of probiotics in improving cytological quality.

Another finding of this study was a significant decrease in the cervical smears showing mildly abnormalities (ASCUS/LSILs) in the women after taking probiotics. Although most of these mild abnormalities will regress without treatment, some may signal a precancerous condition or rarely cancer, especially in those positive for HR-HPV [27, 28]. Verhoeven et al. evaluated women with LSIL-related HPV infections, and they also found a significantly higher cervical smear resolution rate in the probiotic group [19]. Since a LSIL is not a surrogate predictor of cervical cancer and as there was no impact on HR-HPV clearance, there is currently no conclusive clinical evidence of the effect of probiotics on cancer prevention, although in-vitro and animal studies have provided such evidence [29].

Several studies have shown that viral load measurements of HR-HPV types in cervical specimens can be a suitable indicator of viral clearance or persistence [30, 31]. In the present study, we also found that a low viral load was the only independent factor predicting HPV clearance. Although estimation of HR-HPV viral load by HC-II has been shown to correlate well with viral load estimated by real-time PCR [32], multiple infections, cross-reactivity of nononcogenic HPV types, and variability in the cellularity of cervical samples may have biased the results. Therefore, the HC-II assay has only been validated as a semi-quantitative test for HR-HPV viral load measurement.

There are several limitations to this study. First, substantial risk factors for cervical cancer such as smoking, number of sexual partners, and the use of oral contraceptives were not included for analysis, and this may have led to some form of bias. Second, status of BV and vaginal pH, which are thought to be important variables determining HPV clearance, were not evaluated in our study. Third, the sample size was small after adjustment because of slow recruitment and also the follow-up period was short. Fouth, HPV typing and quantitative real-time PCR assays were not applied since viral clearance may differ among various HPV types and the accuracy of quantitative PCR in viral load measurement is greater than that of HC-II.

## Conclusion

We demonstrated that the application of the probiotic strains *Lactobacillus rhamnosus* GR-1 and *Lactobacillus reuteri* RC-14 provided no influence on genital HR-HPV clearance but may have decreased the mildly abnormal and unsatisfactory rates of cervical smears. A larger well-designed randomized study is warranted to confirm these results.

## Data Availability

The data are available upon reasonable request from the corresponding author.

## Abbreviations

ASCUS: Atypical squamous cell of undetermined significance
BV: Bacterial vaginosis
HC: Hybrid capture
HR-HPV: High risk human papilloma virus
LSIL: Low grade squamous intraepithelial lesion
PCR: Polymerase chain reaction
